# Autism Spectrum Disorder Screening Instruments for Very Young Children: A Systematic Review

**DOI:** 10.1155/2016/4624829

**Published:** 2016-12-26

**Authors:** Patricia O. Towle, Patricia A. Patrick

**Affiliations:** ^1^Westchester Institute for Human Development, Valhalla, NY, USA; ^2^School of Health Sciences & Practice, New York Medical College, Valhalla, NY, USA; ^3^Department of Pediatrics, New York Medical College, Valhalla, NY, USA

## Abstract

Research on ASD in infancy has provided a rationale for developing screening instruments for children from the first year of life to age of 18 months. A comprehensive literature search identified candidate screening tools. Using methodological probe questions adapted from the Quality Assessment of Diagnostic Accuracy Studies (QUADAS), two Level 1 and three Level 2 screening instruments were reviewed in detail. Research evidence conclusions were that instrument development was in beginning phases, is not yet strong, and requires further development. Clinical recommendations were to continue vigilant developmental and autism surveillance from the first year on but to use the screening instruments* per se* only for high-risk children rather than for population screening, with considerations regarding feasibility for individual settings, informing caregivers about strengths and weaknesses of the tool, and monitoring new research.

## 1. Introduction

Autism spectrum disorder (ASD), a neurodevelopmental condition that significantly affects social functioning, communication, and patterns of interests and behavior, is most often a life-long disorder that requires extensive educational, vocational, and community support [1, 2]. There is increasing evidence for the efficacy of early intervention [3–5], from which follows the assumption that the cost to society and to families can be mitigated to some degree by alleviating early symptoms and building early skills, leading to reduced symptom severity and greater independence later in life. Early detection based on this reasoning, as well as the high prevalence rates (1 in 68 children [6]), is currently being emphasized from clinical, advocacy, and public health sectors [7–10]. Early detection is increasingly accomplished through systematic developmental surveillance, which is a combined effort on several fronts by several systems and their components. Healthcare professionals are strongly encouraged to understand the importance of developmental screening, to know and inquire about developmental milestones, and to respond to caregiver concerns in specific and actionable ways (referring for more in-depth evaluations, as well as to early intervention systems, continuing to monitor the child) [11–13].

One important component of this general effort is the use of formal screening instruments that focus on specific questions and aid in decision-making about further steps for referral and evaluation. For example, the “Learn the Signs. Act Early.” campaign of the Centers for Disease Control and Prevention's National Center on Birth Defects and Developmental Disabilities (NCBDDD) calls for, in addition to systematic developmental surveillance, autism-specific screening at 18 and 24 months [8]; a similar protocol is endorsed by the American Academy of Pediatrics [14].

Autism screening instruments have been available for this age range since the late 1990s [15]. Currently, there are a number of screeners that apply to children* starting* from age of 18–24 months and up to 30–36 months [11, 16]. The most well-used such instrument in the United States as well as in Europe [17] is the Modified Checklist for Autism in Toddlers (M-CHAT) [18], which has now been revised as the M-CHAT-R/F [19]. The M-CHAT-R/F is considered a Level 1 screener because it is intended to screen at a population level, that is, all children regardless of their risk level for developmental disabilities, including ASD.

There are Level 2 screening instruments for this age range as well. A second-level screener is to be applied to children at risk, such as those who have come to the attention of their parents or pediatrician in order to see if they are more likely to have ASD than another type of delay or disability and in this way route them more efficiently to relatively high-cost comprehensive evaluation procedures. An example of a Level 2 screening instrument with demonstrated predictive validity is the Screening Test for Autism in Two-Year-Olds (STAT) [20, 21], which is intended for children 24–36 months administered by a clinician who first goes through a training program to become a reliable administrator.

Over the last decade, however, several screening instruments have been developed to identify children with autism spectrum disorder* under* the age of 18 months, primarily during the 12–15-month period, sometimes extending down to 6–8 months and sometimes extending to 24 months. Several research, theoretical, and clinical trends have led to interest in developing such tools. In research, there are at least two different relevant areas of research—each demonstrating that symptoms of ASD can be seen quite early in life—that have led to these efforts. The first is the retrospective analysis of home movies of infants and toddlers later diagnosed with ASD, and the second is prospective studies of high-risk (for ASD) children from birth to three years of age. Both of these study methods have shown that a number of autism spectrum symptoms in the social, communication, behavioral, motor, and temperament realms can be seen as early as 12 months and sometimes younger.

Zwaigenbaum and colleagues [22], in a review that included home-movie research studies, concluded that behaviors that consistently differentiated children with ASD versus children who are typically developing (TD) at 12 months of age are the following: less or atypical orienting to people and their faces, reduced responding to name, reduced display of positive affect and social smiling, less eye contact, and less use of gestures used for communication, including pointing. Yet when focusing on studies that utilized a control group of infants with non-ASD developmental disabilities (DDs), a smaller list of behaviors emerged. Two studies found differences at 9–12 months between infants with ASD and DD for response to name and looking at faces (children with ASD showed these behaviors less than children with DDs), but not for frequency and type of gesture [23, 24]. Clearer differentiations between participants with ASD versus DDs have been found as the study age approaches 24 months [22]. In addition, there are also important distinctions between groups of infants that have differential timing patterns of symptom emergence. These retrospective home-movie analyses helped to confirm that some children with ASD have relatively typical first-year social/linguistic development and then change during their second year, while others show a prodromal phase of autistic symptomology during their first year, some by at least six months of age [25]. The early-home-movie method of inquiry has made it clear that an appreciable subset of children who will go on to be diagnosed with ASD will show social-communication and behavioral deficits and differences by the end of their first and beginning of their second year of life [22, 26].

The second influence was from the consortium of “infant sibling” studies, wherein newborn siblings of children diagnosed with ASD are enrolled for study prospectively. The rationale for this approach was that a genetics-based etiology would result in a higher incidence of ASD in these young children than in the population at large and thus would be an efficient means of studying the very early development of ASD. In fact, Ozonoff and colleagues [27] documented that the recurrence rate of ASD for these high-risk children was nearly 20%, with male gender and more than one older sibling diagnosed with ASD conferring additional risk.

Several study sites within the infant siblings consortium specifically track early social communication, social interaction, and other types of developmental behaviors so that possible patterns of very early emerging symptoms in these behavioral areas could be documented for those eventually confirmed as having ASD at age of 3 years [28]. These multisite studies began in the early 2000s and thus there are several reviews that document early social-communicative difference and presence of various repetitive behaviors [29–32]. The reviews report that very few behavioral differences could be seen at the 6-month mark, but it is from 6–9 months on to 24 months that the skill levels in key areas diverge for those infants later diagnosed with ASD versus those who were not [30–33]. The behaviors that distinguish these two groups from 12 months on were in fact very similar to those coded in the home-movie studies—reduced shared positive affect, social responsivity, communicative gesturing, and response to name [31, 33–35]. While significant group differences were found, that does not mean that every single infant who was later diagnosed with ASD had these early symptoms. Similar to the home-movie studies, there were subgroups based on timing of symptom emergence [31]. Landa and colleagues [36] demonstrated that some infants eventually diagnosed with ASD (at three years of age) maintained more typical social skills up until about age of 14 months (slightly over half, 16/30) and then began to lose them, compared to a group who was fairly symptomatic at 14 months (slightly less than half, 14/30).

The increased understanding about very early emergence of autism spectrum symptoms has led researchers to theorize about primary and secondary causes of delay and disorder in ASD, in order to concurrently elucidate etiology and suggest directions for intervention. A number of studies, both within the infant siblings general paradigm and apart from it, have focused on investigating the earliest postnatal neurologic and information-processing divergences from typical development such as in the areas of eye tracking, attention to faces, attentional regulation to objects versus social stimuli, visual and auditory processing, brain-based sensory integration systems, and interconnectivity within cerebral cortex [37]. Documenting very early brain organization and information-processing differences underpins frameworks for understanding such as the “developmental cascade,” that is, earlier foundations that support continued development and, if disturbed, lead to failures of, or atypicality in, later developmental capabilities [38, 39]. For example, Chawarska et al. [40] demonstrated differences in attention to faces in 6-month-olds who would go on to receive an ASD diagnosis and hypothesized that “A limited attentional bias towards people early in development is likely to have a detrimental impact on the specialization of social brain networks and the emergence of social interaction patterns.” In fact, a number of studies have demonstrated significant disturbances in fundamental social engagement precursors and processes during the first year of life, as studied through eye-tracking paradigms, fMRI, behavioral tasks, and EEG protocols [39].

Experts who advocate “the earlier, the better” point to these findings as well as three other tenets of early intervention, that is, the neuroplasticity of the developing brain and the associated notion of critical periods, that is, that there are functional and timing/phase-bound periods of rapid development when intervention is optimized [41, 42], and the interactive-specialization hypothesis—the widely held theory that children's brains and capabilities develop through the ongoing, iterative transaction of neuromaturation with environmental experiences [43, 44].

Therefore, the findings that observable* behavioral* ASD markers become more evident from about 12 months on (see above) can be viewed as both the result of earlier, derailed neurocognitive processes and also a disturbed foundation that may produce further ASD symptoms. For example, since children's early learning language and gesture learning is highly dependent upon frequent, ongoing communicative exchanges with an adult, then a child who is not interested in faces, does not easily become socially engaged, and is not responsive to language input is very vulnerable to delay in language acquisition as well as in development of social cognition and conventional play skills [45, 46]. In addition, the rapid emergence of language and social communication from 9–18 months constitutes its own critical period for these specific skills [47].

It is in this context that researchers have become very interested in a downward age extension in autism-specific screening tools that can be applied clinically. Earlier detection means earlier intervention and the possibility of preventing the secondary effects of atypical development, in other words, a more fully developed autism profile. There has been some success with screening instruments that target children from 18 to 24 months of age and older [22–25]. The goal of this paper is to review the extant screening instruments for children under 18 months of age.

To be included in the review, the screeners needed to have peer-reviewed published reports of sensitivity and specificity from prospective study designs. The instruments' development, method of administration, reliability and validity, receiver operator characteristics, and feasibility of use are described. The review also seeks to draw conclusions regarding methodological and substantive challenges highlighted by this close examination, recommended use of such screeners, and directions for future research.

## 2. Methods

### 2.1. Literature Review

This more focused review is part of a larger review conducted for the New York State Department of Health Clinical Practice Guidelines for Autism Spectrum Disorder for children ages birth to three years. For this larger, evidence-based review of autism screening instruments, a set of search terms were developed and refined and then applied to a broad set of databases such as PsycInfo and Medline. The resulting 2,188 abstracts returned were then sorted through for articles that met the inclusion criteria set for the review. The target was screening instruments in peer-reviewed journals, published in English, and that had reported receiver operator characteristics (ROC) or performance measures of sensitivity and specificity and/or positive predictive and negative predictive values.

Adequate ROC are considered a definitive test of predictive validity and utility for screeners because they inform about percentages of individuals that will be detected or missed by the screening procedures. Other ways of assessing the validity of tests use statistics applied to groups based on probability theory and can produce highly significant results but still have a relatively low effect size or account for a relatively small amount of variance. Therefore, significance can be high, but precision may still be low.

Once the screeners were identified, the authors did search for all the peer-reviewed articles that were relevant to the development, reliability, and validity of the instruments. This way each one could be critically evaluated in terms of its development, the constructs measured, psychometric properties, and performance. Examining these details yielded insights into methodological issues that will be important to consider as attempts to identify very young children with ASD continue.

### 2.2. Features of Studies Testing the Predictive Validity of an ASD Screening Instrument

Studies are conducted differently depending on whether the instrument is intended to be a Level 1 (population level) or Level 2 (for high-risk children) screener. How this affects the recruitment, inclusion criteria, and number of participants is covered in the sections below. Nonetheless, every study compares the screening results to a reference standard or “gold standard,” which consensus dictates to be the true test of whether the child actually has the condition or not. For autism spectrum disorder, this invariably entails a “Best Estimate Diagnosis” by an experienced practitioner who is drawing from a variety of information gathered about the child (e.g., history, caregiver interview, standardized tests, and direct observation of the child).

When a child fails a screening test, he/she is shown to be at increased risk for the condition, and the result is called positive. When the child passes the screener, the result is called negative; the child is not considered at increased risk for the condition. The screener results, characterized as positive or negative, are then compared to the reference standard, which is also determined as positive or negative for each child. When a child is positive for the condition on the screener and is shown to have the condition on the reference standard, then it counts as a true positive. If the child did not turn out to have the condition, then it was a false positive. The negatives follow in the same fashion.

Sensitivity (Se) and specificity (Sp) are calculated with proportional formulae using true and false positives and negatives. In explanatory terms, Se represents the degree to which the screener accurately detects the condition. The measure runs from 0 to 1.0, with 1.0 being perfect detection. However, the predictive validity of the screener is only understood by considering both Se and Sp together. Sensitivity can be very high if the screener has included almost everyone, and in doing so, of course, it included children with ASD. Specificity represents the extent to which the screener distinguished the targeted condition from other or no disabilities. Therefore, Sp balances out the Se by showing that it did not include too many extra children who in fact had a different developmental disorder or had no developmental problems at all.

Acceptable levels of Se and Sp depend on the outcome or condition of interest. More specifically, for the detection of a preventable communicable disease, investigators may tolerate lower specificity (greater proportion of false positives) for higher sensitivity (greater proportion of true positives). Nevertheless, it is suggested that the threshold values for acceptable levels of sensitivity and specificity should be at least .80 or greater, although accuracy levels of .90 or above are considered optimal [48–50].

Positive predictive value (PPV) is a measure that reflects the percentage of children who screened positive and who did actually have the condition based on the gold standard testing. Negative predictive value (NPV) is the inverse—the percentage of children who screened negative and who did not have the condition. Positive and negative predictive values are directly related to the prevalence of the condition under study within the population; these measures are not intrinsic to the instrument. In other words, a screening instrument that has high sensitivity and specificity may have low PPV if the prevalence of the condition is low—a positive result is less likely to be accurate if a condition is rare.

The ideal procedure for examining the predictive validity of a screening instrument involves a direct route between screener administration and diagnostic outcomes, with the most knowledge available about scoring outcomes for every child who was given the screener. This can be challenged by attrition during the various phases of the study, and, as will be seen, many studies include additional steps and criteria for a child to advance from one phase of screening and testing to the next. After thorough review of the initial group of studies, several were excluded from in-depth reporting because of this feature; they were no longer considered a test of the targeted screener because other, sequential procedures obscured the findings.

### 2.3. Types of Screeners (Administration)

ASD screening tools (or any type of behavioral screeners) generally take two forms: a caregiver-rated checklist or a clinician observation. A variant is to have the clinician administer the checklist to the parent.

### 2.4. Screening Instrument Reviews

For this review, each screening instrument was described and then critiqued in the following way. The extant literature on the instrument is summarized in the Background section. In the next sections, the instrument's development, how it is administered and scored, and the constructs it measures are described. The Research Summary section follows an adaptation of the Quality Assessment of Diagnostic Accuracy Studies (QUADAS-2) [51]. This research quality assessment method follows a typical evidence-based procedure whereby experts are gathered, probe questions from a pool are chosen for a given diagnostic or screening question/tool, the questions' utility is field tested, and reliability of ratings is established. The QUADAS and QUADAS-2 are very widely used for both diagnostic and screening tests in both the behavioral [52–54] and medical [55–57] fields. This rating system was not applied formally (i.e., assigning numerical ratings) but instead adapted in the following way: (1) the four domains of Participants, Index Test (the screening tool), the Reference Standard (the “gold standard”), and Timing and Flow were used as units of review with the additional domains of Evaluation and Performance; (2) probe questions were developed for each of the domains; and (3) the probe questions were applied to each study and conclusions were summarized.

Table 1 shows the probe questions developed for this review and their significance. For the Participants domain, different questions are needed for Level 1 versus Level 2 instruments. The differential parameters are discussed more in depth in the Results section. For the Screening Instrument domain, it is important to note if there is anything about how the instrument was administered in the study that would differ from how it would be used in the community. Examples are provided in the table and in the results.

For the Reference Standard domain, the extent to which the participants each had face-to-face evaluations using Best Estimate Diagnosis as well as how this was supported with standardized procedures and other disciplinary evaluations was noted. As part of the inclusion criteria, only studies that used Best Estimate Diagnosis based on DSM-IV or DSM-IV-TR [58] criteria were included. A second very important question was whether or not those who conducted the diagnostic evaluations were blind to the screener status of the children, that is, if they were screen negative or positive, since expectation bias could be introduced in this way. Finally, it was important to note what outcome categories the study considered, so that information about ASD severity level of children and differentiation from other types of disabilities could be known.

The Timing and Flow domain has a number of features. Two important questions—was the screener done prospectively and was the time interval between the screening and diagnosis adequate—were not included because conditions were met for all studies reviewed. The probe question used refers to conditions moving children from screening to diagnosis that could obscure interpretation directly from the screening tool being examined. It was this probe question that led to understanding that a number of the studies had multistep screening protocols without sufficient analysis of Se and Sp for each step, and therefore such studies were eliminated from more in-depth critique.

In addition, two other probe questions were used to evaluate the results* per se* of the instrument. The first was what developmental characteristics of the children identified as having ASD were presented. This will allow a comparison across screeners for which children were detected in terms of overall developmental level. The second referred to what extent the false positives detected other types of disabilities.

### 2.5. Methodological and Content Themes

As the screening studies were reviewed, themes were extracted related to methodology and content issues. Finally, conclusions were drawn as to recommendations for use, themes regarding this body of research as a whole, and recommendations for future research and development of these types of screeners.

## 3. Results

### 3.1. Level 1 Early Screening Instruments for Autism Spectrum Disorder

#### 3.1.1. Features of Autism-Specific Level 1 Screening

Level 1, or first-level screening, is meant to be applied at a population level—for example, to all children coming through a pediatrician's or family physician's office. These are “low-risk” or “unselected” samples. In developmental surveillance parlance, however, the role of Level 1 screening is to first identify children who are at risk for any developmental disability, which may include ASD as well as other types. Another term for this type of screener is “broadband.” In the case of autism-specific screening, the intention is to identify those at risk for ASD specifically, but on a population level. Regardless, the intent to screen populations has implications for feasibility characteristics of screeners, for tolerance limits for levels of Se and Sp, and for research methods.

Feasibility refers to practical features related to cost, time, and ease of administration, scoring, and interpretation. Therefore, one expects a Level 1 screener to be quick and low cost so that it can fit into well-child visits as easily as possible. In terms of performance, there is somewhat more of an emphasis on Se rather than Sp, so that as few as possible cases are missed. Thus, there is more latitude for lower Sp, as long as it is close to being acceptable (>.80).

There is a particular set of methodological hurdles to conducting research for screening instruments at a population level, which involves screening thousands to tens of thousands of children. The first hurdle is actually screening this large a number of children, but just as challenging is following up enough of the children to find out who did have ASD or did not. After the initial screening, researchers typically call the parents/caregivers to invite them to come in for an evaluation that will take time and effort on the family's part. Participation is voluntary, so there may be a bias for families who are motivated to come in for further evaluation. Usually quite a bit of attrition occurs at this step that lowers the percentage of the population sample examined with the “gold standard” compared to those who were screened. If the investigators have basic demographic information on those who did not come in for the next phase, the best they can do is test if there are any significant differences between those who consented to move on to a full evaluation and those who did not on some baseline or demographic variables.

Most investigators focus first on getting positive screens (families/participants) to come back for fuller evaluation, since at least PPV can be calculated. But the final hurdle is that children who screened negative may be in the tens of thousands, and thus it is virtually impossible to examine them all directly. Even taking a proportional sample is difficult because of the lack of motivation for parents to come in for a longer evaluation if they do not believe their child is at risk. Of families that do consent, there may be a bias for families with more financial resources, or where one parent does not work, and/or higher education on the part of these volunteer participants. The overall result is that “true” Se and Sp can rarely be estimated because of these attrition and sampling problems.

Level 1 screening instruments, therefore, are often* developed* by using smaller samples and involving relatively high numbers of children who do have ASD. This is necessary to determine which items discriminate among children with ASD, DD, and TD. Yet, the screener's validity as a Level 1 instrument cannot be known unless it is then tested on a large, low-risk, unselected sample. Obviously, such studies are expensive and labor-intensive, and even focused resources and efforts often cannot overcome the barrier of uneven participation.

One important discovery from this review was the extent that studies attempting to describe the predictive value of a given screener were actually describing a screening program with multiple phases and instruments rather than a more pure test of efficacy of one screener. Ideally, the screener is given, and as many children as possible are followed through to the diagnostic testing phase, and thus there is a relatively direct route between the screener and the diagnostic outcome. In the case of three fairly well-known instruments—the Early Screen for Autistic Traits (ESAT) [64, 65], the Checklist for Early Signs of Developmental Disorders (CESDD) [66], and certain studies with the Infant-Toddler Checklist (ITC) [59, 67]—the children were filtered through prescreens or two-stage screening without explication of the effect on potential true and false positives and negatives on the targeted screener. Based on the probe questions under the “Index Test” and “Timing and Flow” domains, these flaws were considered sufficiently problematic to exclude as studies of the ROC of the instruments. On the other hand, the studies are often able to provide PPV estimates, have other merits, and provide results that are informative about many features of the screening process. Thus, some are reviewed in the preliminary narrative for the instrument.

Table 2 presents first-level ASD screening instruments that have peer-reviewed papers reporting Se and Sp or PPV and NPV, as well as the studies that have been excluded once reviewed in depth.

#### 3.1.2. Level 1 Instruments Excluded from Detailed Review

The* Pervasive Developmental Disorders Screening Test (PDDST-II)* [68] is a parent-report instrument for children ages 12 to 48 months. It is the only instrument that has three screening levels to it. However, the only ROC presented are in the manual, and there is no paper describing such a validity study published in peer-reviewed journals.

The* Early Screen for Autistic Traits (ESAT)* [64, 65], which targets 14-15-month-olds, is often reviewed as an early ASD screener, but it does not yet have peer-reviewed articles that report prospectively obtained Se and Sp for its intended age group. Dietz and colleagues [64] reported on its development and utilized retrospective parent reports to discover which items discriminate ASD from non-ASD. Swinkels and colleagues [65] further described validity testing and did apply the ESAT prospectively, giving results in terms of percentages and odds ratios, but not ROC.

As important, interpretation of the studies for this screener is limited by the two-phase protocol used for the ESAT. Specifically, first a four-item screening test was given to parents of children being screened, and it was only those who failed this prescreen (by failing any one or more of the four items) who were then given the ESAT. Although the ESAT's predictive value was tested in reference to children diagnosed with ASD, we do not know its performance if given as a first-level screener; it cannot be known who was left behind when the 4-item prescreening was given in terms of true and false positives and negatives. The study is really describing a screening program that the ESAT is part of, not the predictive value of the ESAT.

Oosterling and colleagues [69] compared ESAT to prediction with the Social-Communication Questionnaire (SCQ) [70] and Infant-Toddler Checklist [71], but the sample was older (primarily 18–24 months) than what the screener was originally developed for. Finally, Dereu and colleagues [72] included the ESAT in a screening study where children were first identified to be at risk through childcare staff rating the Checklist of Early Signs of Developmental Disorders [66]. Because the ESAT was given in a second phase along with several other screeners and many of the children were not in its intended age range, this report was not considered to reflect the predictive ability of the ESAT.

There are also feasibility concerns about the measure in that it is actually administered by a trained psychologist through parent interview. In the validity study, the psychologist was evaluating the child through play and a cognitive assessment at the same visit and making judgments on each item, overriding the parent if he or she expressed no concern about a given behavior when the psychologist did have concern. This is a protracted process for a screening instrument, one that approaches an abbreviated evaluation, and thus would not seem practical for most settings.

The studies of the ESAT do yield important information about the screening process (e.g., they found that the caregiver/parents had a very low rate of agreement with the psychologist about their child's symptoms when the child actually was shown to have ASD later). Further studies addressing these methodological issues would be required to substantiate the ESAT's use as an ASD screening instrument. It would also require a translation if not used in Dutch-speaking countries.

The* Checklist for Early Signs of Developmental Disorders (CESDD)* [66] is a daycare staff-rated checklist for children 3 to 36 months. There are at least two promising innovations embodied in this instrument, the first being its use of childcare staff as informants, given their familiarity with the children they care for and their presumed knowledge about child development. For the study, the staff were given training sessions about early symptoms of ASD as well. The second is that the checklist included slightly different sets of items depending on the age of the child, which helped in accommodating developmental changes over the age range.

The CESDD studies share interpretive challenges with the ESAT in that the tool was part of a two-phase screening process, with the CESDD as the first-phase screener. After a child was flagged as at-risk based on the CESDD, then parents filled out several more screeners and only advanced to the evaluation phase if their child then failed one of these additional screeners, a set of language items, or the MacArthur-Bates Communication Inventory [73]. Finally, a child could also go on to the full evaluation if parents requested it out of concern. Also, the attrition was high at this phase—only 31% of the families went on to Phase 2. By the time the children were evaluated for ASD, they no longer represented a group that had been determined to be at risk only by the CESDD. Therefore, although the Se (.80) and Sp (.94) were good to excellent, it is not clear how much these statistics represent the CESDD alone. Finally, the CESDD has only been used in the Dutch language.

#### 3.1.3. Level 1 Instruments Included for Detailed Review

*(A) Infant-Toddler Checklist (ITC)*

*(1) Background*. The ITC is a 24-item caregiver-report checklist for children ages 8–24 months. It is part of the Communication and Symbolic Behavior Scales Developmental Profile [71]. The CSBS-DP was developed out of an earlier, longer version—the CSBS [74]. This instrument, which is for children 6–24 months of age, was originally developed for detection of early language delays, not ASD specifically. However, with its emphasis on prelinguistic communication, including social components such as eye gaze and emotion, it became clear that the instrument targeted key early features of ASD and was addressing younger children than many other instruments. There are three parts to both the original and the Developmental Profile version: a short, parent-rated ITC; a longer parent-rated Caregiver Questionnaire (CQ), which is an elaborated version of the ITC and includes some of the same questions; and the Behavior Sample (BS)—a semistructured clinician observation and rating procedure—that was shortened considerably (to 20–30 minutes) for the Developmental Profile version so that it could in fact function as a screener. At this point only the ITC has sufficient published research as an ASD screener.

The first published research on the ITC focused on its function as a language screener and in the context of using all three of the CSBS components as a child progressed through first- and then second-phase screening and then diagnostic evaluation [75]. In 2004 and 2008 the ITC, combined with other components of the CSBS-DP, was investigated as an autism spectrum disorder screener. The Wetherby and colleagues study [76] is not described here because most of the children were 18–24 months when screened, making the study more similar to those for ages 18+ months.

Wetherby et al.'s study [67] also was not reviewed using the probe questions because the study children went through a three-stage screening procedure wherein the CSBS-DP Behavior Sample and then the Systematic Observation of Red Flags (for autism) (SORF) further narrowed the group who were eventually diagnosed with ASD. This study is nonetheless reported on in the Research Summary section because parts of the study are very informative (see below).

More recently, the instrument was reported on in terms of its utility when used with children with a mean age of 12 months [59], again as a broadband screener.

*(2) Instrument Development*. The original choice of items for the CSBS and therefore for the CSBS-DP is noteworthy because they were informed by important foundational research on normal development of prelinguistic social communication [77, 78]; this essential knowledge about when and how children develop a range of communication strategies within an interactive caregiving context helps us understand how typically developing children bring their own capacities to engage the caregiver. It is through this engaged relationship, ongoing interaction, and the resulting perpetual feedback loops that children develop social-linguistic competence [79].

*(3) Measurement Strategy, Constructs Measured, and Scoring*. The ITC is a 24-item checklist that can be filled out by a very familiar caregiver in about 5 minutes. The ITC has been normed so that it can be used with cutoff scores (both the checklist and its normative tables for cutoff scores are free downloads) and, with the use of the purchased manual, will yield standard scores as well.

Because the ITC was originally developed as a language screening measure, it focuses almost exclusively on social and communication behaviors. It does include four items on object use or play. There are no items about repetitive behaviors, unusual sensory reactions, or temperament issues. (It was conceived as a screener that would lead to further evaluation, most specifically by the CSBS Behavior Sample, and it is from the Behavior Sample that repetitive behaviors are scored, using the SORF.)

In contrast to many other such checklists, the ITC yields a number of subscale scores in addition to a total score. The Social Composite consists of three subscales:* Emotion and Eye Gaze* (3 items),* Communication* (4 items), and* Gestures* (5 items). The Speech Composite includes* Sounds* (3 items) and* Words* (2 items), and the Symbolic Composite contains* Understanding* (2 items) and* Object Use* (4 items).

Given these subscales, failing the screen can be done in more than one way: if the child is below the cutoff on either the total score, or the Social Composite, or the Symbolic Composite. The category denoting a positive screen is “Of Concern.” Scoring the ITC relies on a normative table with cutoff scores to classify as follows: (1) typical skills: readminister in 3 months; (2) elevated risk: monitor and readminister in 3 months; and (3) at-risk: refer immediately for further evaluation. Standard scores are also obtainable using the purchased manual.

In addition to these scores used as cutoff points, it appears that a “fail” also occurs when the parent checks off the single item “have a concern about your child.”

*(4) Research Summary.* Wetherby and colleagues [67] went to great lengths to locate every child from the original ITC screening sample (*N* = 5,385) who was diagnosed with ASD at age of three years or older, finding them either through their own research program, through follow-up mailings to families, or through autism treatment programs in the catchment area. Sixty children with confirmed diagnoses were then compared to children with other DDs and TD children. Of the 60, 56 had at-risk ITC scores at some point before 24 months. At 6–8 months, only 20% of the children scored at-risk, but 77% did so at 9–11 months. However, for the 12–14-, 15–17-, 18–20-, and 23–24-month age groups, the percentages of at-risk scores ranged from 91% to 100%.

The study by Pierce and colleagues [59] is the only study reviewed in depth here because it met the criteria of including ROC analyses and did not involve two-stage screening (see Table 3). The authors tested the ITC as an autism screener when given to parents through pediatric practices at the one-year well-child checkup. The mean age the ITC was filled out by parents was 12 months, with a range of 10 to 15 months. Screening 10,479 children, they did have 1318 “fails,” with an at-risk rate of 12.5%. For practical reasons, the researchers were only able to follow up and examine directly 184 (14%) of those children who screened positive for ASD. Of those failing the ITC between the ages of 10–15 months, 75% were found to have ASD, DDs, or LD (language disorder) at age of 3 years (i.e., PPV = .75). Twenty percent were diagnosed with ASD (PPV = .20). Therefore, ITC was again found to function best as a broadband screener.

*(5) Conclusions.* The ITC has the advantage of being a short parent checklist that can be applied in community settings and can identify children as young as 12 months as being at risk for developmental delays including ASD, language delay, and other delays, but it does not successfully distinguish ASD from other DDs. The authors have recommended it as a broadband screener, therefore, and suggest its use more as a continuous monitoring device over the second year of life. There is obvious utility in detecting other disabilities that require follow-up and attention. For the children with ASD, outcome developmental quotients demonstrated the presence of children with a range of functioning, including some children with average intellectual functioning.

The main methodological issues of this study were as follows: (1) it is not known if the evaluators were blind to the participants' screening status; and (2) the attrition from their original set of over a thousand children scoring at-risk was very high. Other studies on the ITC, including those with Se and Sp reported, were excluded because their methods did not allow for straightforward interpretation of the ITC's predictive power given that children were moved through to definitive evaluations for reasons unrelated to the ITC. This instrument has only reported PPV, which is appropriate because all children from screening sample could not be followed up to know the proportion of true and false negatives. The ITC would need further studies that reported Se and Sp using more conventional screening validity methods in order for more conclusive judgments to be made about its utility.

*(B) First-Year Inventory (FYI)*

*(1) Background*. The FYI is a 62-item caregiver-rated checklist meant for screening 12-month-olds. It was first produced in 2003 and remains an unpublished instrument. Its development and first applications were described in Reznick et al. [80] and Watson and colleagues [81]; a recent study focused on an analysis of what combinations of items were most predictive [82]. Turner-Brown et al. [60] reported Se and Sp on a community, low-risk sample. A shortened version (the FYI-Lite) has been reported upon [83], but no specificity and sensitivity estimates were produced. The development of the instrument is currently ongoing (Baranek, pers. comm.).

*(2) Instrument Development.* The authors chose target behaviors for items based on an extensive review of the literature that included retrospective studies, case studies, studies of home movies, and prospective studies from the Baby Sibling laboratories as well as from community programs and from videotapes of infants from the authors own laboratory. They obtained feedback from experts and parents on the item wording and eventually piloted the instrument with a community sample of parents. The final version of 63 questions is obtainable from the authors.

Reznick and colleagues [80] reported on a normative community sample that was gathered by a mass mailing to families who had an infant child based on birth records. Of the 5,941 sent out, there was a return rate of 25%. Parents filled out the questionnaire when their child was within a month of 12 months old. This paper described how the subscales of the FYI were developed based on the parent responses from this sample (see section below).

Watson and colleagues [81] used the population sample to test the ability of the FYI to discriminate among children who had been diagnosed with ASD, typically developing children, and children with non-ASD developmental disabilities by having parents of older children (but under 5 years of age)* retrospectively* rate their children at 12 months. They examined both the total risk scores and the risks scores for each of the eight constructs. Analyses of variance and post hoc analyses showed highly significant differences across the three groups, with the total score and most of the constructs showing an ability to discriminate among children with ASD, DD, and TD.

*(3) Measurement Strategy, Constructs Measured, and Scoring*. This is a parent-rated questionnaire with 63 items; the items have several different response types. The authors considered their items covering the broad categories of “social-communication” and “sensory-regulatory functions” and purposely included some items indicating “generalized developmental delay” because of their increased association with autism.

Forty-six of the items are rated on a four-point scale from “never” to “often.” Fourteen of the items revisit key questions but have the parent choose a frequency rating (e.g., “some of the time” to “all of the time”). There is one item asking the parent to circle the consonant sounds their child makes. The final two items are open-ended questions about concerns about development and other medical issues the child may have.

The authors then conducted a construct shaping procedure that yielded eight constructs under the two following domains: (1) social-communication domain—Social Orienting and Receptive Communication, Social-Affective Engagement, Imitation, and Expressive Communication; and (2) sensory-regulatory domain—Sensory Processing, Regulatory Patterns, Reactivity, and Repetitive Behavior.

Risk scores were developed by examining frequency distributions of items to find those of low endorsement but high atypicality; these were identified across the eight constructs, standardized to account for the different number of items within constructs, and a cutoff score determined for a combined or total risk score (cutoff = 17).

*(4) Research Summary. *Turner-Brown and colleagues [60] followed up the normative sample of 1,192 nonselected families who had filled out the FYI when their child was approximately 12 months old and agreed to be contacted after their child was three years old. A letter was sent out that included the Social Responsiveness Scale (Preschool) and a developmental questionnaire that inquire about developmental status, any early intervention, and any diagnoses. A total of 699 families returned the questionnaire.

Children whose parents' responses on these measures indicated, based on a set of score cutoff criteria, a high risk and/or possibility of ASD were invited to come for an in-person evaluation. Twenty-eight children were seen for a clinical evaluation. Between in-person assessment and parent report of community diagnoses, 9 or 1.3% of the 699 children were found to have ASD at age of 3.

Using a cutoff total score of 19.2, the PPV was .14. Investigators report that 4 out of 9 children identified with ASD met the total score cutoff (or 44% sensitivity) and that the majority of children (665/690) who did not have ASD at age of 3 screened negative (or 97% specificity). They also examined ROC using a two-domain cutoff. This yielded an improved PPV (.31) with very similar Se (.44) and Sp (.99).

*(5) Conclusions. *It would appear that the FYI is still in its formative stages. It has shown some ability to predict ASD from the early age of 12 months, but the sensitivity is unacceptably low for an ASD clinical instrument; thus further research is needed to increase its utility. This is one of the only studies of its kind that made an effort to ascertain the developmental status of the entire sample (who sent back questionnaires at age of three, *n* = 699). Over half of the ASD false positives had other developmental problems, which increases its utility as a developmental screener. Although their developmental outcome information was not extensive, there appeared to be a range of abilities including some very low and some high.

Its authors suggest that the 62-item length may be too long for routine office screening and an FYI-Lite version has been used in two papers (without Se and Sp information reported). They report on their research website that they are continuing to work with this screening tool.

#### 3.1.4. Level 1 Conclusions and Recommendations

For this young age range, two screening instruments were examined after the criteria of published performance information and interpretable “timing and flow” filtered several others from consideration. Both instruments have demonstrated some success at detecting ASD under 18 months but actually function better as broadband screeners that detect other disabilities as well as ASD. The ITC identifies itself as such a screener, while the FYI endeavors to be autism-specific. Both were able to detect children under age of 18 months who had a range of developmental levels at age of three years, but both are also in need of continued research to demonstrate efficacy. The FYI appears to be in process of revision, as well.

### 3.2. Level 2 Early Screening Instruments for Autism Spectrum Disorder

#### 3.2.1. Features of Autism-Specific Level 2 Screening

Level 2 screeners are intended to distinguish ASD* per se* among children already identified as being at high risk for a number of developmental disabilities and delays. In the field of autism screening, there are added nuances in that high risk is conferred in three major ways. The first involves children that have already been identified as being at risk, but this is operationalized in a variety of ways as well: children who have been referred to evaluation clinics and have appointments to be evaluated thoroughly, children who have failed an autism-specific screening instrument through parent report or clinician administration in a primary care or research setting, and children for whom their caregivers or primary care providers have expressed concerns. The second way is risk status unique to a relatively recently developed research paradigm: they are younger siblings of children that have been diagnosed with ASD, since the “Baby Siblings” research has shown the prevalence to be significantly increased among them [27]. A third way, not addressed in this review, is by a child having a congenital (preterm status) [84, 85] or genetic condition (e.g., Fragile X, Down syndrome, or Angelman syndrome) that is associated with increased risk of an ASD diagnosis [86, 87].

As with Level 1 screeners, the goals for Level 2 have implications for research and feasibility. Validity research designs typically compare identified groups of children with ASD and other DDs and frequently a TD group. Ideally the comparison groups are matched on gender, age, and developmental level. In terms of sensitivity and specificity, the latter becomes important because of the explicit effort to distinguish ASD from other DDs. In terms of feasibility, the length of administration may be longer, and need for training may be present because of this more advanced phase of identification.

Table 4 presents second-level ASD screening instruments that have peer-reviewed papers reporting Se and Sp or PPV. They are presented in order of most recent publication (which reported Se and Sp) date.

#### 3.2.2. Level 2 Instruments Excluded from Detailed Review

The* Autism Observation Scale for Infants (AOSI)* [88], a clinician observation instrument, was developed by a group of Canadian researchers who are part of the prospective infant sibling studies consortium. They were interested in developing a standardized measure for early autism symptoms for children as young as 6, 12, and 18 months. They developed the protocol in the early 2000s in response to this need and began to generate published reports in 2005. However, by 2014 the authors did not recommend it for clinical use based on inadequate sensitivity and specificity [89].

The AOSI is a semistructured interactive measure of 19 items that takes about 20 minutes to be administered by a clinical professional. The clinician must be trained in the AOSI and, in addition, needs to be quite familiar with infant behavior as well as young children with ASD.

The items reflect many of the social-communication behaviors identified through both basic studies of the development of ASD and the development of other infant screening instruments. Its creators also included early information-processing behaviors, such as the child's ability to shift visual attention away from one stimulus to another. The AOSI also includes some items reflecting temperamental features, such as atypical reactivity (under- and overreactive to environmental input).

The AOSI represents an intensive effort to take advantage of the infant sibling research protocol to reveal very early emerging behaviors of ASD and to create a manageable semistructured observation protocol to use in both research and clinical settings. At this point more work is needed to refine procedures to stabilize reliability coefficients and to realize adequate sensitivity and specificity.

#### 3.2.3. Level 2 Instruments Included for Detailed Review

*(A) Screening Test for Autism in Two-Year-Olds (STAT)*

*(1) Background*. The STAT is a clinician-administered instrument for children 12–36 months and takes about 20 minutes to administer. It was developed in the late 1990s, with reports on development, reliability, validity, ROC, and community applications and feasibility from 2000 to the present. Although it was created for children from 24 through 35 months, there is one study that investigated its utility for children 12–24 months [61], and the authors are continuing to examine scoring strategies that address earlier detection (Stone, pers. comm.).

*(2) Instrument Development*. The development sample was described in a brief report by Stone and colleagues [20], and the results were considered preliminary evidence for the instrument's utility. They had a very small sample: 7 children with autism and 33 with DDs and/or language impairment (LI), all between 24 and 36 months. The participants were given the STAT while attending a clinic for a full evaluation because of developmental concerns. The authors developed the scoring and the cutoff scores and then applied it to another set of high-risk children to achieve Se of .83 and Sp of .86 for Autistic Disorder (AD) only.

Further development of the STAT was described in Stone and colleagues [21]. Groups of children referred for evaluation were given the STAT at the same time as their comprehensive evaluations at a university clinic. In Study 1, 26 children who were diagnosed with AD were compared to 26 children who were diagnosed with developmental delay and/or language disorder in order to establish cutoff scores for adequate sensitivity and specificity. They determined that a cutoff score of 2 resulted in Se of .92 and Sp of .85. In Study 2 of this paper, a similar procedure was used to look at other features of validity and reliability, but no ROC coefficients were reported. This time 50 children had AD and 39 had DDs and/or LI. All three of the above studies specified that the screening was intended to detect AD and not the milder presentation of Pervasive Developmental Disorder-Not Otherwise Specified (PDD-NOS), as referenced to the DSM-IV [58].

Chiang and colleagues [90] adapted the STAT to a Taiwanese version. These authors made some item substitutions, so their instrument would be considered an adaptation. They conducted the study in the same way that the Stone articles described and obtained an Se of .93 and Sp of .74, predicting to AD only and excluding PDD-NOS.

*(3) Measurement Strategy, Constructs Measured, and Scoring*. The STAT is a semistructured play-based interactive tool that takes about 20 minutes to complete. It can be given in less time if the child fails sufficient items to reach the at-risk cutoff score before all items are given. The clinician does have to be trained in order to administer it, and this involves an investment of time and cost. There is an online tutorial that takes several hours to complete and includes a reliability scoring test that must be passed in order to obtain the training certification. Vanderbilt University does conduct training workshops, including specialized ones for MDs. The information is available at http://vkc.mc.vanderbilt.edu/vkc/triad/training/stat/physicians.

There are four domains of two-to-four items each: Play, Requesting, Directing Attention, and Motor Imitation. In fact, the items are probing for social communication, joint attention, pretend play and functional object use, and motor/gestural imitation. There is an emphasis on social communication in this scale and there does not seem to be an opportunity to directly account for highly atypical behaviors (although this is reflected to some degree through* lack* of pretend and functional use with objects in this tool).

For each item, the child receives a Pass, Fail, or Refuse, using the child's best performance for up to three trials. Only the total score is used for determining at-risk status.

*(4) Research Summary*. The only study to be considered here is the one that looked at children 12–24 months [61], since it falls within our age inclusion criterion. Participants were younger siblings of children diagnosed with ASD (*n* = 59) and children referred for developmental concerns (*n* = 12). They had the STAT administered from 12 to 24 months and were evaluated for ASD at 24 months (see Table 5). Although for studies using high-risk children between 24 and 36 months the cutoff score was 2, it was adjusted for the study using children between 12 and 24 months. Performance measures were generated for a higher more optimal cutoff score, 2.75, as follows: Se = .95, Sp = .73, PPV = .56, and NPV = .97. The authors noted that prediction improved at 14 months and thus recalculated the measures after excluding the 12-13-month-olds, with the following results: Se = .93, Sp = .83, PPV = .68, and NPV = .97.

*(5) Conclusions*. The STAT is a clinician-administered, semistructured interactive screener that shows adequate to strong prediction up to this point. It requires an investment of time and money to train front-line providers; however, once this is accomplished, presumably the administrator has a skill set that facilitates identification and referral for ASD and DDs independent of the actual screener application. Although it was originally developed for children between age of two and three years of age, this one paper has shown in a preliminary way its utility for children between one and two years. One weakness in this study was that the sample size was relatively small (group with ASD ended up being 19, and typically developing group was 12). Another issue is that the children were evaluated for ASD at 24 months of age. A minority of children will change their diagnosis between 24 and 36 months, and thus it would be important to have a study that also evaluated the children at 3 years of age for more precise prediction coefficients. The original papers restricted the STAT to AD, but the study about younger children extended the prediction to ASD with good ROC. One important contribution was that prediction to later diagnosis was examined in for the youngest versus older children. Across a 12-month age range, starting at age of 12 months, prediction improved starting at 14 months. Fifty percent of false positives had developmental problems warranting evaluation and intervention. Based on the relatively high developmental quotients at outcome diagnosis, this instrument is detecting a group of children that includes those with milder levels of disability. The authors continue to refine its structure and scoring for future reports (Stone, pers. comm.).

*(B) Parent Observation of Early Milestones Scale (POEMS)*

*(1) Background.* The POEMS, a 61-item caregiver-rated checklist, was developed relatively recently by researchers in Canada who recruited, through an Internet site, families with infant siblings of children diagnosed with autism spectrum disorder. They were interested in tracking the emergence of ASD features to facilitate earlier identification in these high-risk children. Papers in 2012 and 2015 described development, reliability, and validity of the measure [62, 91]. The 2012 paper had ROC reported.

*(2) Instrument Development. *The researchers developed items based on established measures of symptoms of ASD in young children, such as the ADI-R [92] and the CARS [93].

*(3) Measurement Strategy, Constructs Measured, and Scoring. *This is a caregiver-report checklist, but in the one published study parents filled out the POEMS online and on paper and then sent it through the mail or reported it over the telephone (the parent and examiner both had a copy and the examiner recorded the parent responses).

The items represent a broad range of behaviors that are known to characterize young children with ASD, including those outside of the core behaviors. For example, the items addressed mood regulation, sensory responses, visual tracking, and motor skills. More specifically, the authors describe the items addressing the following types of behaviors: early social and communication skills or deficits, restricted interests, ritualistic, repetitive, nonfunctional behaviors, intolerance to transitions and waiting, difficulties with new foods, loud noises, sleeping, and toileting, problems with attention and visual tracking, and problems with motor agility and movement. Each item was scored on a 1–4 scale, with 1 representing typical and nonproblematic behavior and 4 representing extreme difficulty. Each item has its specific description for the low and high behavioral anchors. Only a total score is obtained; no subscales scores have been developed.

*(4) Research Summary. *Feldman and colleagues [62] recruited families with an older sibling diagnosed with autism and then followed up younger, infant siblings with parents filling out the POEMS multiple times, at least a month apart. They followed up 108 children. Data analyses focused on scores they had for groups of both HR/ASD and HR/No ASD at 3, 6, 9, 12, 18, and 24 months. To get the high-risk siblings diagnosis by 36 months, they relied on parent report of community diagnoses. They were able to complete the ADI-R on ~70% of the HR/No ASD sibs (*n* = 99) and to confirm they did not have ASD. They were only able to give the ADI-R on 3 out of the 9 HR/ASD children (see Table 5). At 12 months, Se was .71 and Sp was .68; at 18 months, Se was .89 and Sp was .65. The overall PPV was .21.

*(5) Conclusions. *The POEMS is a medium-length parent checklist (61 items) for very early ASD detection. Although Se and Sp did not both reach ideal levels (>.80) at any age, it was the best at 18 months, with Se at .89 and Sp at .65. Given that Se was under acceptable levels until 18 months, the checklist does not offer an advantage for children below 18 months of age. It is noteworthy that the Se and Sp were around .70 at 12 months of age, given the difficulty of detecting ASD specifically at this early an age through a parent checklist.

For the sake of developing the measurement tool, the authors gave the checklist every three months, but presumably a choice would be made as to the ideal time to use the instrument for screening. However, the repeated parent reporting used in the instrument's development also may have served to heighten parents' observational skills, thus increasing veridicality of their reporting, and this would not be the case for one-time administration. The POEMS is a recently developed measure with one published study and future work may serve to increase the Se and Sp levels.

*(C) Autism Detection in Early Childhood (ADEC)*

*(1) Background. *The ADEC is a clinician-administered play interaction tool with 16 items for children 12–36 months. It was developed by a researcher in southern Australia. The development and initial examination is reported in a 2007 in-house publication [94]. In 2010, Hedley et al. [95] translated the instructions to Spanish and showed good Se (.94) and Sp (.92–100) for a sample of 19–36-month-old children. Subsequent papers were published in 2014 [63, 96], the latter reporting ROC. In 2015, a paper with ROC was published that used a sample from the US [97], but it focused on children from 18 months on. Importantly, the intent as specified in the first of these studies was to distinguish AD only. In the 2015 paper the authors changed the outcome category to ASD.

*(2) Instrument Development. *Behaviors were identified from retrospective parental reports [98] and video analysis [99].

*(3) Measurement Strategy, Constructs Measured, and Scoring. *The ADEC is a clinician interaction/observation instrument with 16 items that takes 10–15 minutes to administer.

The specific behaviors that are observed during the administration of the ADEC are response to name, imitation, ritualistic play, joint attention and social referencing, eye contact, functional play, pretend play, reciprocity of smile, reaction to common sounds, gaze monitoring, following verbal commands, delayed language, anticipation of social advances, nestling, use of gestures, and task switching.

As an example of scoring,* social response *has been operationalized as whether a child responds to his or her name when called by the examiner over five trials. If the child responds in the first or second attempt, he or she is scored a 0; in three to five attempts, he or she is scored a 1; and a 2 is given if the child does not respond to his or her name in any of the five attempts.

Response scores for each item range from 0 (*appropriate*) to 2 (*inappropriate*), with a possible maximum score of 32. Based on Se and Sp data provided in the manual, a score of 0–10 indicates a low risk for AD, 11–13 a moderate risk, 14–19 a high risk, and >19 a very high risk.

The training for ADEC is not formal. A video is supplied with purchase of the manual, and the clinician is expected to view it, study the manual, and practice until proficient. The ADEC website (https://shop.acer.edu.au/autism-detection-in-early-childhood-adec) specifies that the individuals qualified to administer the ADEC are “Master degree in a health profession (e.g., speech pathology, occupational therapy, special education, or social work) OR Bachelor degree in a health profession, PLUS evidence of training in assessment, OR ACER Specialist Certification.”

*(4) Research Summary. *Nah and colleagues [63] recruited families with children 12–36 months from a variety of sources over a several-year period in order to accumulate a varied group of 70 children diagnosed with AD, PDD-NOS, and other DDs and as TD. They first had research assistants administer the ADEC, and then the children and parents returned for a full evaluation, using Best Estimate Diagnosis, including the ADOS. This study calculated performance measures using both matched and unmatched samples. For the matched sample, they selected children from two groups (AD and other DDs) that matched on NVIQ and Vineland Adaptive Behavior Scales Adaptive Behavior Quotient (VABS ABC). The unmatched group retained all the children that ended up in their diagnostic groups (AD and other DDs) but the groups had significantly different mean NVIQs and Vineland ABCs. The authors predicted from ADEC failure (cutoff score of 11) to outcome, but only to AD versus other DDs, leaving out the PDD-NOS group; this strategy will increase the Se and Sp. They also calculated measures with and without combining the other DDs and TD group (see Table 5). For all the comparisons, Se was 1.0, and Sp ranged from .74 to .90. In the unmatched sample PPV was .84 and NPV was 1.0.

*(5) Conclusions. *The ADEC is a clinician-administered structured play interaction tool. Se reached 1.0; however, investigators specifically removed milder children and this can inflate performance measures compared to studies that include the continuum of severity. Its outcomes need to be considered in the context that the measure was intended to detect Autistic Disorder and not milder presentations. Another issue was that it appeared that concurrent diagnoses were made to validate some very early screening ages. Therefore, if some of the children were diagnosed at 24 months or less, then there is a chance that some would change diagnosis by age of three. Sp was in the high 70s (.74 unmatched sample and .77 matched sample) and was thus just under the acceptable level (>.80). Discriminating ASD from DDs was accommodated through a matched sample and performance remained essentially the same.

Although the authors intend for the instrument to be used by clinicians with limited training, the description of the instrument suggests that to score the items correctly the administrator would have to study the directions, including examples of passing and nonpassing behaviors, and practice with this tool to some extent before being able to administer it quickly. In the most recent paper, a more protracted training period was described.

#### 3.2.4. Level 2 Conclusions and Recommendations

Three second-level screening instruments were reviewed that have a beginning research base. Two are clinician-administered tools. The STAT shows promise but needs continued study with larger subject samples. It has feasibility considerations in that there is time and cost for initial training; the training itself does confer the added value of a nonspecialized clinician understanding early ASD symptoms in greater depth. The ADEC is building a research base with adequate Se but Sp that is less so, and the research on the younger age continuum of its targeted population is just beginning. The ADEC requires study and practice on the part of the clinician but this training is less formal and resource-intensive than the STAT. One parent-report checklist, the POEMS, has a very beginning research foundation. Its current length may preclude widespread community use except in the case of children who are at higher risk for ASD and thus for whom the expenditure of extra time is warranted.

## 4. Discussion

### 4.1. Interpretation Challenges: Methodological Themes Associated with Study Design

After comparing these early screener studies, several essential method issues emerged that need to be addressed in order to create more consistent and comparable future screening studies for very young children.

#### 4.1.1. Inclusion Criteria

The first is that important features of participant inclusion/exclusion are either not specified or inconsistent. Two of the five studies do not describe any neurodevelopmental features of either those initially included or those evaluated apart from developmental levels at the point of reference standard evaluation. The STAT authors [61] did specify that significant sensory, motor, genetic, and metabolic disorders were conditions for exclusion. Preterm status is another relevant participant characteristic; the FYI study excluded children born preterm because the researchers wanted the child to be 12 months of age and not have adjusted age complicating that goal. However, preterm children have been shown to be at higher risk for ASD [84, 85] so it is also relevant to the degree of risk present in the sample.

#### 4.1.2. Outcome Categories

A second issue is inconsistency in the outcome categories when the reference standard diagnostic testing is performed. Both the STAT and the ADEC originally stated that the intent was to detect more severely affected children (AD and not PDD-NOS) but in the most recent articles changed that designation to ASD. The studies in general are variable in their designation of non-ASD disability detected. Some have clear criteria based on standardized tests for language disorder and developmental disorder, whereas others use more vague terms such as “broad autism phenotype” and “developmental concerns” [59]. While it is important to accommodate different levels of possible developmental delays and differences in the outcomes, consistency in definition will be important.

#### 4.1.3. Developmental Level of Children at the Outcome Stage

This is also an important piece of information because it gives insight into whom the screener detected—higher versus lower functioning individuals, for example. There is considerable inconsistency across studies regarding this information. There is the complication that some children will not be able to respond to standardized tasks in order to achieve a score so that there will usually be children not represented by such scores.

### 4.2. Constructs Encompassed in Very Early Screening Instruments

Clearly, different approaches have been taken to construct measurement in the screeners. The ITC, for example, was developed as a social-communication measure and does not include repetitive behaviors (although play items function to some extent when typical milestones are not endorsed).  Table 6 shows the different symptom domains that were derived from comparing the screeners and the items that represented them. The most common differences are the extent to which regulatory/temperament items (difficult to soothe, cannot tolerate waiting, and gets upset easily) and developmental items (language, motor, and play milestones) are included apart from the core social-communication and repetitive, restricted behavior symptom domains. Brian and colleagues [100] found that items related to temperament (difficulty with transitions and reactivity) at 18 months did discriminate well between high-risk infant siblings children diagnosed with ASD or not at age of three years and that these features added independent prediction from social-communication items.

A similar result was reported by the authors of the POEMS. When items were analyzed that were most predictive from early months to ASD diagnosis at age of three years among high-risk biologic younger siblings, “problems with waiting” was as strong as any of the social and communication items [91]. These results suggest that temperament and reactivity items may be important to include along with core symptom items, but the extent to which developmental items add predictive value has yet to be examined.

The AOSI authors attempted to add a very early neuropsychological feature to prediction. Although the screener has not been shown to be predictive at a level for clinical use at this time, it is noteworthy that they included items that are related to certain early differences that are pursued in more laboratory-based studies.

### 4.3. Challenges to Early Detection of ASD before 18 Months of Age

By sampling children from below 12 months up to 18–24 months, some of these very early screening studies were able to demonstrate that prediction was less stable the younger children were. Wetherby and colleagues [67] showed that of children diagnosed with ASD at 36 months fewer attained at-risk scores on the Infant-Toddler Checklist below 12 months (6–8 and 9–11 months), but from 12 to 24 months the percentages identified as being at risk (after serial screening) ranged from 91% to 100%. Wetherby and Prizant [71] examined concurrent validity by correlating ITC scores with clinician ratings on a structured play procedure tapping social-communication competence and found the correlations increased with age as follows: 6–11 months, .57; 12–17 months, .78; 18–24 months, .87. Stone and colleagues [61], testing the STAT as a screening tool for children 12–24 months, found that the number of false positives was much higher for the 12-13-month groups (38%) compared to 14–17 months (13%) and 18–23 months (11%) [61]. For the POEMS, Feldman and colleagues [62] reported that sensitivity improved and only received acceptable levels for the instrument as children were 18 and 24 months, compared to 3, 6, 9, and 12 months of age.

There are a number of reasons why these youngest children are more challenging to identify. They range from measurement challenges to features intrinsic to very young children who develop symptoms in different patterns in terms of timing and clinical presentation. These issues are considered as follows.

#### 4.3.1. Difficulty with Parent Report for Early, Subtle Behaviors

Starting with the measurement methods themselves, many rely on caregiver report, asking them to make judgments on behaviors that are undoubtedly subtle at the ages up to 18 months. While parents and other consistent caregivers have the advantage of the greatest familiarity with and interest in their children, under certain circumstances parent report is not as reliable as direct observations [61, 101]. In a study comparing parent observation to detailed behavior counts from home videos, Ozonoff and colleagues [102] found that, while some aspects of the parent reporting were accurate, their reports regarding slowing of their child's development and gradual loss of skills were not consistent with video observations. The authors speculated that skill loss during this earliest period—between 12 and 18 months—may have been too gradual for parents to notice. During administration of the ESAT, wherein a trained psychologist was observing the child at the same time the parent was reporting on symptoms, it was common for the psychologist to override the parent judgment in the direction of more autism symptoms for the child [65]. Wetherby and colleagues' study [67] found that while parents were reporting a number of atypical symptoms, they often indicated that they did not have any concern, that is, regard them as problematic. Since several studies have found 18 months to be the average age of first parent concern for children diagnosed with ASD [103], it is possible that lack of language, underdeveloped social skills, and any unusual play patterns are more evident to them by the middle of the second year compared to earlier.

#### 4.3.2. Timing Patterns of Symptom Emergence

Different timing patterns of symptom emergence among children with ASD make the ages before 12 months to 18 months particularly ambiguous. Retrospective parent-report, systematic observations from home movies and Baby Siblings consortium prospective studies have all been used to characterize the timing and nature of symptom development in ASD. Initially, mainly two trajectories had been described: early onset, wherein slow development is noted from about six months onward with gradual unfolding of more frank ASD symptoms after the one-year mark and throughout the second year, and later onset, often thought of as regressive autism [25]. In the latter pattern, parents reported that their child developed normally in all areas for the first year and then lost language and social skills in the second year, with this often becoming noticeable to them between 18 and 24 months. In contrast, more recent studies have endorsed three general patterns—(1) early onset, (2) relatively intact first-year development with loss of skills in the second year, but generally earlier than parents report [102], and (3) a “plateau” pattern, wherein first-year development is normal but soon after 12 months the child stops making progress and eventually manifests a full ASD profile by 24–36 months [22, 104]. It is likely, therefore, that for patterns (2) and (3) the first half of the second year may be a time of transition and ambiguous behavior.

One of the most important timing distinctions gleaned from prospective studies is that reported by Landa and colleagues [36]. Using a Baby Siblings prospective sample, approximately half the infants who would eventually be diagnosed with ASD showed symptoms at 12 months of age, but the other half was not detectable until after 14 months. Clearly, this distinction has important implications for attempts at very early screening. The differential prediction patterns of the STAT [61] and the ITC [67] reflect this symptom emergence timing.

The symptom domain of repetitive and restricted behaviors appears to present specific challenges in terms of timing and thus detection under 18 months. Although several studies have shown that certain types and rates of repetitive behaviors will distinguish groups of children with ASD versus DDs versus TD by 12 months [105–107], there is also evidence that repetitive behaviors as a whole may emerge or become more evident as the child approaches 3 years of age and older. For example, Moore and Goodson [108] reported the increase in repetitive behaviors from 2 years, 10 months to 4 to 5 years of age. The type of repetitive behavior plays a role; this effect was attributable in part to emergence of circumscribed interests and unusual preoccupations, symptoms that may not be observable at much younger ages. Guthrie et al. [109], using the ADOS-T, demonstrated this pattern but starting at early ages: restricted and repetitive behaviors became more prevalent in the second half of the second year, continuing through 30–46 months.

#### 4.3.3. Phenotypic Variability

Phenotypic variability also complicates very early detection at these youngest ages. Children and individuals with ASD are known to vary widely in their presentation, and there are a number of sources of this variability. First, there is a difference in degree of variability between the two core symptom domains. Whereas social communication is always delayed and different in children with ASD, the RRB symptom domain has been observed to be more variable in children under three years [110], in addition to its tendency to increase in evidence over the second and third years of life.

Variability in degree of overall severity is also a known feature of ASD, and since young children with milder symptoms are more difficult to diagnose definitively under 24 months of age, it stands to reason that the less precise screening measures also are not as effective for early detection. With a Baby Siblings sample, Ozonoff and colleagues [111] reported that of those who were diagnosable at 36 months less than half (47%) were diagnosed at 18 months, and only 60% could be diagnosed at 24 months. These results represent a subgroup of the population of children with ASD, however (high-risk younger siblings). Guthrie and colleagues [109], using a community sample, showed that only a small proportion of their community sample had an unclear diagnostic presentation before 24 months and thus required follow-up for diagnostic clarification at three years of age.

The new designation of “specifiers” for the DSM-5 diagnostic criteria [112] encourages distinction between children with different cognitive levels, severity levels of the two symptom domains, and degree of language impairment. The earliest presentation of children with ASD and also with what will be enduring cognitive and language disability may be particularly difficult to differentiate from children with global delays. On the other hand, children with ASD but who are not inherently intellectually delayed and whose language is relatively intact may represent the more mildly affected children, who tend to be missed by ASD screeners in general and thus most likely by the very early screeners as well [3, 19]. In fact, all reviewed studies used DSM-IV criteria and thus it will be important to observe what impact the somewhat more conservative DSM-5 criteria will have on the predictive power of early screeners.

In addition to differences in the timing of symptom emergence, recent studies have begun to include other developmental domains, such as fine and gross motor, language, and nonverbal cognitive skills, when characterizing patterns of developmental disturbances. This approach generally yields more classes or models of trajectories [33, 113].

All told, these results suggest that prediction from behaviorally based screening instruments before 12 months to 14 months will be inherently unstable. From about 14 months on, more accurate prediction to later diagnosis is achievable. One important methodological recommendation that can be made with confidence is that when this age group is included in screening studies with older children, separate calculations must be made for the younger participants. If the participant sample includes children up to 24 months and even older, resulting ROC may overestimate prediction from the youngest group.

## 5. Conclusion

### 5.1. General Recommendations for Autism and Disability Screening Studies

Table 7 summarizes methodological recommendations for future early screening studies gleaned from all phases of this review, ranging from summaries from the general literature on disability screening as well as autism-specific screening to those generated from the probe questions adapted from the QUADAS system for evidence-based analysis of diagnostic tests, to conclusions gleaned from these specific studies.

### 5.2. Recommendations for Clinical Use of Very Early ASD Screening Instruments

It is important to consider what clinical practice recommendations can be made from the current evidence. In fact, the evidence base for these early screeners is just being developed and therefore cannot be considered strong. Most of the instruments focused on in this review do have more than one paper that describes development and establishes different features of reliability and validity; all of them have one paper with ROC coefficients for children less than 18 months, as well. Most of these studies have some shortcomings in terms of research quality or else can be viewed as one of several needed to build a case for efficacy.

Given the current strong interest in the earliest identification of ASD, it is likely that more studies will soon add to the evidence base, and there remains a strong rationale for their continued development and conservative use. The growing body of studies around early neurological and information-processing disturbances and their possible link to early emerging behavioral disturbances offer much promise for interventions that interrupt the “developmental cascade” that leads from a prodromal autism pattern to a fully developed ASD presentation [42, 45]. The early screeners reviewed are a starting point for this endeavor and offer the opportunity to monitor children at risk for ASD; in addition, all were shown to detect other developmental delays at about a 50% rate of their false positives. These advantages can be seen to outweigh concerns about overidentification. Another reasonable concern by practitioners may be whether intervention services are available for such early-identified children. Importantly, any child with a significant developmental delay can be referred, evaluated, and possibly provided services by the state-administered public early intervention program. However, for autism-specific services, there is recent emerging documentation for some success with children who are symptomatic at 9–15 months [3, 46], and this will likely continue to be a focus of practice and research.

Recommendations for their use first need to be put in the context of the larger effort of early developmental surveillance for all types of disabilities including ASD. This should always be conducted via checking progress on developmental milestones and/or using an evidence-based broadband developmental screener, for example, Ages & Stages Questionnaire, knowing the red flags for developmental delay and early autism behaviors and being responsive any time a caregiver brings up a developmental concern. The recommendations regarding formal screening instruments are necessarily slightly more conservative, given the current practice climate calling for efficient, evidence-based instruments. The recommendation, therefore, is to use one of these instruments at these early ages only if a child falls into one of the risk categories: an older biologic sibling who has been diagnosed with ASD; a child who has already failed a developmental screening, one who has shown behaviors or whose parents have reported behaviors of concern to the health professional; or a child whose parent has expressed a concern over general development or ASD* per se*. The following are also considerations for use. First, each agency or practice may have its own priorities for resource expenditure and instrument administration in light of their capability and clinical flow. Second, the strengths and limitations of the screener should be understood and shared with caregivers. Third, rescreening and monitoring for developmental delays and autism symptoms should take place if results are equivocal and in fact are built into the intended administration strategy of some of the tools. Finally, ideally data can be collected using a sound prospective design or program evaluation approach to add to our knowledge base for the instruments.

Clearly the value of such early detection is to support the family to obtain early intervention as soon as possible, and there is a growing base of evidence for the effectiveness of early intervention. Very relevant to this review, there are now reports about intervention efforts for high-risk children detected with ASD symptoms early in the second year [3, 16, 105]. Given the potential consequences of not acting to build early skills and remediate barriers to more effective cognitive and social learning (more costs to society, less independence and access to full community participation by individuals), acting early with what is available seems prudent.

## Figures and Tables

**(a):** 

Domain	Level 1 screener probe questions	Level 2 screener probe questions
Sample/participants	*Was the sample appropriate in size and scope? *	*Was the sample appropriate in size and scope? *
	*How representative was the sample?*	*Did investigators use a sample matched for developmental level?*
	*Were there exclusion criteria based on other disabilities?*	

**(b):** 

	Level 1 and 2 screener probe questions
Screening instrument	*Was there anything about how the screener was administered that would be different from its intended use in a nonresearch, community setting?*
	*Were there any issues regarding the way it is scored in the study?*

Reference standard	*Did all children receive a BED from in-person evaluations? How extensive was the information available to the clinician making the Best Estimate Diagnosis? *
	*Were the reference standard evaluators blind to the screener risk status of the children?*
	*What diagnostic outcome categories were used to test prediction from screener to reference standard?*

Timing and flow (including attrition)	*Was there excessive attrition through any phase of screening and evaluation?*
	*Were there conditions besides attrition that filtered the negative and positive screens from the original screening to the reference standard diagnostic testing phase?*

Evaluation	*How were performance/predictive values calculated?*
	*Was performance/prediction for younger versus older children explored?*

Performance	*What were the performance/predictive values?*
	*What was the developmental level of children detected?*
	*Of the false positives for ASD, what proportion had other developmental or learning disabilities?*

**(a):** 

Screener	Dates of paper(s)	Country developed	Ages (mos)	Strategy
Infant-Toddler Checklist (ITC)^†^	2004 2008 2011^*∗*^	US	8–24	Caregiver checklist
First-Year Inventory (FYI)	2007 2013^*∗*^ 2014	US	12	Caregiver checklist

**(b):** 

Excluded from in-depth review	Dates of paper(s)	Country developed	Ages (mos)	Reason for exclusion
Pervasive Developmental Disorders Screening Test (PDDST-II)		US		No peer-reviewed articles (ROC or otherwise)
Early Screen for Autism Traits (ESAT)		Netherlands	14-15	No prospective studies done in age range; two-stage screening
Checklist for Early Signs of Developmental Disorders (CESDD)	2010 2012	Netherlands	3–40	Two-stage screening
Infant-Toddler Checklist (ITC) (2 of 3 studies)	2004 2008	US	8–24	Studies involved two-stage screening

^†^The ITC was evaluated for ASD prediction and the authors concluded that it functions best as a broadband screener, identifying children at risk for disabilities, a proportion of whom will be diagnosed with ASD.

^*∗*^This paper was reviewed in detail for research methods because it was an ROC study.

**Table 3:** Research summaries for the Infant-Toddler Checklist (ITC) and First-Year Inventory (FYI).

Probe questions	ITC [59]	FYI [60]	
Sample/participants			
*Was the sample appropriate in size and scope?*	Low-risk community sample; large to start with (10,479), but attrition was high for the reference standard evaluation phase; 184 at the end; 14% of high-risk sample.	Yes, population study; mailed to almost 6,000 and got a 25% return rate. 699 filled out developmental and ASD screening questionnaires after child's third birthday.	
*How representative was the sample?*	These parameters were not reported.	Although the sample was diverse, there were a disproportionate number of Caucasian and highly educated families responding to later phases of the screening study.	
*Were there exclusion criteria based on other disabilities?*	They specified that no exclusion criteria were exercised for either the population sample or the follow-up.	It was specified that children born preterm were excluded.	

Screening instrument			
*Was there anything about how the screener was administered that would be different from its intended use in a nonresearch, community setting?*	No.	No.	
*Were there any issues regarding the way it is scored in the study?*	Note that the ITC can be failed in four different ways—low score on either or both of two subscales, total score; there may be differences in true and false positives given the source of fail criterion.	The authors explored predictive validity based on several different ways of using subscales scores and total score.	

Reference standard			
*Did all children receive a BED from in-person evaluations? How extensive was the information available to the clinician making the Best Estimate Diagnosis?*	Cognitive, ADOS-T, and ADI-R; children seen every 6 months up to three years of age. They evaluated children every 6 months and gave “at-risk” dx's of ASD from 12 to 18 months, “provisional” dx's from 19 to 31 months, and established dx's from 32 to 36 months with ADI-R. Five children with provisional dx's no longer had dx at the last evaluation.	Mixed—some children brought in for Best Estimate Diagnosis including all information, ADOS, and occupational therapy evaluation (*n* = 9). Three others were determined to have ASD based on diagnostic evaluations submitted by parents. Those evaluations all used the ADOS.	
*Were the reference standard evaluators blind to the screener risk status of the children?*	Not reported.	Yes.	
*What diagnostic outcome categories were used to test prediction from screener to reference standard?*	ASD, LD, DD, and no diagnosis. LD and DD defined by Mullen Scores, “other” by parameters such as motor delay.	ASD, other DDs' diagnosis, or treated through EI services, developmental concerns (no diagnosis but concerns), and no concerns.	

Timing and flow			
*Was there excessive attrition through any phase of screening and evaluation?*	Out of 10,479, there were 1316 fails. Out of those, only 346 were referred for testing by the researchers, with a list of practical reasons why the others might have been missed. Out of 346 they lost another 232 for a variety of reasons, so in the end they worked with 184 high-risk children plus 41 TD children referred as a comparison group.	No.	
*Were there conditions besides attrition that filtered the negative and positive screens from the original screening to the reference standard diagnostic testing phase?*	No.	No issues—they were able to make some assessment of developmental status of all 699.	

Evaluation			
*How were performance/predictive values calculated?*	They combined ASD with other DDs to calculate PPV because they were considering the ITC a broadband screener.	They were not able to see the FYI negatives in person but did have parents report diagnoses, EI services, developmental concerns, and two parent-rated screening questionnaires for DD and ASD symptoms.	
*Was performance/prediction for younger versus older children explored?*	Screened at 12–15 months. But their breakdown showed that diagnosis was less stable at 12–18 months and became more stable towards 24 months.	N/A—all screened at 12 months.	

Performance			
*What were the performance/predictive values?*	PPV = .75 for all disabilities. PPV = .20 ASD alone.	*Total score* PPV = .14. NPV = .99. Se = .44. Sp = .97.	*Two-domain cutoff* PPV = .31. NPV = .99. Se = .44. Sp = .99.
*What was the developmental level of children detected?*	IQs ranged widely but did include higher functioning children: MSEL Composite (M = 100; SD = 15). M = 78.6. SD = 17.5. Range = 49–106.	Sample size includes higher-functioning children but difficult to characterize because 9 children had ASD and only 6 had Mullen Composite scores; four were average or higher and two were very low. M = 94.7. Range = 62–127.	
*Of the false positives for ASD, what proportion had other developmental or learning disabilities?*	69.7%.	65% (who met total score cutoff). 85% (who met two-domain cutoff).	

ITC = Infant-Toddler Checklist; ASD = autism spectrum disorder; FYI = First-Year Inventory; BED = Best Estimate Diagnosis; ADOS-T = Autism Diagnostic Observation Schedule-Toddler Module; ADI-R = Autism Diagnostic Interview-Revised; dx = diagnosis; LD = language disorder; DD = developmental disability; EI = early intervention; TD = typically developing; PPV = positive predictive value; NPV = negative predictive value; Se = sensitivity; Sp = specificity; MSEL = Mullen scales of early learning; M = mean; SD = standard deviation.

**(a):** 

Screener	Dates of paper(s)	Country developed	Ages (mos)	Strategy
Screening Test for Autism in Two-Year-Olds (STAT)	2000 2004 2008^*∗*^	US	12–24	Interactive clinician observation
Parent Observation of Early Markers (POEMS)	2012^*∗*^	Canada	3–24	Caregiver checklist
Autism Detection in Early Childhood (ADEC)	2014 (2)^*∗*^ 2015	Australia US	12–36	Interactive clinician observation

**(b):** 

Excluded from in-depth review		Country developed	Ages (mos)	Reason for exclusion
Autism Observation Scale for Infants (AOSI)		Canada	6–18	Studies eventually showed inadequate Se and Sp.

^*∗*^This paper was reviewed in detail for research methods because it was an ROC study.

**Table 5:** Research summaries for the Screening Test for Autism in Two-Year-Olds (STAT), Parent Observation of Early Milestones Scale (POEMS), and Autism Detection in Early Childhood (ADEC).

Probe questions	STAT [61]	POEMS [62]	ADEC [63]	
Participants				
*Was the sample appropriate in size and scope? *	Small: 19 had ASD. All participants were of high risk because of diagnosed older sibling.	All participants were of high risk because of diagnosed older sibling.	All participants were of high risk based on being scheduled for an evaluation at a specialty clinic.	
*Did investigators use a sample matched for developmental level?*	No.	No.	Yes.	

Screening instrument				
*Was there anything about how the screener was administered that would be different from its intended use in a nonresearch, community setting?*	No.	The POEMS was filled out by families every three months. Giving the POEMS many times could sensitize parents to ASD behaviors, especially since they would already be so because of their older child with ASD. However, this is consistent with its intended use within this study.	The team administering the screener received some training.	
*Were there any issues regarding the way it is scored in the study?*	No.	No.	See evaluation domain below.	

Reference standard				
*Did all children receive a BED from in-person evaluations? How extensive was the information available to the clinician making the Best Estimate Diagnosis? *	Yes; the information available included ADOS and Mullen.	No direct examination for diagnostic status. They relied on parent report of community diagnosis. They were able to give ADI-R to 3 out of the 9 children with ASD to confirm.	BED with cognitive assessment, ADOS, and ADI-R if AD or PDD-NOS diagnosis considered. “77.5% had an independent confirmatory diagnosis from either two other independent professionals who were recognized by the state's autism association or other medical professionals such as pediatricians and psychologists.”	
*Were the reference standard evaluators blind to the screener risk status of the children?*	Not reported.	Most likely, considering they were clinicians in the community who were independent of the study.	Yes.	
*What diagnostic outcome categories were used to test prediction from screener to reference standard?*	Autism, PDD-NOS, DD, LI (language impairment), BAP (Broader Autism Phenotype), and no diagnosis.	Only categories were ASD versus no-ASD. This is a departure from most studies, which also include other DDs. This appeared to be a function of the study methods, which involved using reports the parents obtained from the community.	For initial analyses, ASD (AD + PDD-NOS), other DDs, and TD. However, authors indicated that the ADEC is intended to detect Autistic Disorder, so PDD-NOS was left out for ROC analysis and this suggests that it will detect more severe children on the spectrum.	

Timing and flow				
*Was there excessive attrition through any phase of screening and evaluation?*	No.	No, sample size was adequate.	N/A.	
*Were there conditions besides attrition that filtered the negative and positive screens from the original screening to the reference standard diagnostic testing phase?*	No.	No, sample size was adequate.	N/A.	

Evaluation				
*How were performance/predictive values calculated?*	The groups were combined as follows: autism and PDD-NOS were all ASD; the others were non-ASD. With this categorization, the most false positives were found for 12-13-month-olds, so Se and Sp were calculated both with and without them. They achieved acceptable Se and Sp levels by raising the cutoff score compared to that for the 24–36-month-olds.	Predictive validity was first explored by forming two groups: infant siblings who were confirmed to have ASD at age of 36 months (*n* = 9) and those who were not (*n* = 63). They then compared how the POEMS score diverged over the different age levels.	Investigators left the PDD-NOS group out and compared AD to Other Developmental Disabilities (ODD) with and without the TD group. This can inflate performance compared to studies that include milder children.	
*Was performance/prediction for younger versus older children explored?*	Yes—reported false positives for three different groups between 12 and 24 months. More false positives for the 12-13-month group than older children.	Yes—see below. The sensitivity got higher as age progressed over 3, 6, 9, 12, 18, and 24 months. Sensitivity reached the acceptable level at 18 months.	Yes, for 12–24 versus 24–36 with no differences found. Did not look at the youngest children (under 18 months).	

Performance				
*What were the performance/predictive values?*	Using a cutoff of 2.75: Se = .95, Sp = .73, PPV = .56, NPV = .97. Excluding the 12-13-month-olds: Se = .93, Sp = .83, PPV = .68, NPV = .97.	A cutoff score of 70 resulted in a mean sensitivity (across all age groups) of .74 and specificity of .87; PPV overall was .21. At 12 months, Se = .71 and Sp = .68. At 18 months, Se = .89 and Sp = .65.	Using a cutoff score of 11.	
			*Unmatched* AD versus ODD: Se = 1.0; Sp = .77. AD versus ODD + TD: Se = 1.0; Sp = .89.	*Matched* AD versus ODD: Se = 1.0; Sp = .74. AD versus ODD + TD: Se = 1.0; Sp = .90.
*What was the developmental level of children detected?*	The sample included higher functioning children; at mean of 24 months, MSEL Early Learning Composite: M = 93.5; SD = 23.3.	Not reported.	Not reported.	
*Of the false positives for ASD, what proportion had other developmental or learning disabilities?*	50% of false positives had other DD diagnoses.	Not reported.	10/70.	

STAT = Screening Test for Autism in Two-Year-Olds; POEMS = Parent Observation of Early Milestones Scale; ADEC = Autism Detection in Early Childhood; ASD = autism spectrum disorder; BED = Best Estimate Diagnosis; ADOS = Autism Diagnostic Observation Schedule; ADI-R = Autism Diagnostic Interview-Revised; AD = Autistic Disorder; PDD-NOS = Pervasive Developmental Disorder-Not Otherwise Specified; DD = developmental delay; LI = language impairment; BAP = Broader Autism Phenotype; TD = typically developing; ROC = receiver operator characteristics; Se = sensitivity; Sp = specificity; ODD = Other Developmental Disabilities; PPV = positive predictive value; NPV = negative predictive value; MSEL = Mullen scales of early learning; M = mean; SD = standard deviation.

**Table:** (a) Level 1 ASD screening instruments

Social communication		Repetitive behaviors	Sensory	Developmental	Reactivity/temperament	Atypical information processing
Communication	Social					
*Early Screen for Autism Traits (ESAT)*						
Eye contact		Has interest in different toys	Reaction to sensory stimuli			
Reacts when spoken to	Emotions understandable	Varied play	Likes cuddling			
	Facial expression	Stereotyped movements				
	Attracts attention					
	Brings/shows objects					
	Interest in people					
	Smiles directly					

*Checklist for Early Signs of Developmental Disorder (CSEDD)*						
Lack of eye contact		Shows stereotyped behavior	Dislikes touching and cuddling	Does not explore, passive		
Turns to name	Social smile	Lack of variable play^*∗*^	Unusual sensory behavior	Lack of functional play^*∗*^		
Seems to be deaf	Shares enjoyment	Lack of functional play^*∗*^		Babbling at 12 months	Overreaction to change or other events^*∗*^	
Gestures	Imitation	Unusual postures		Single words at 16 months		
Follows point	Prefers aloneness	Overreaction to change or other events^*∗*^		Spontaneous two-word phrases at 24 months		
Points	Shows object for social bid			Loss of language		
Instrumental communication/hand-leading	Flat affect					

*Infant-Toddler Checklist (ITC)*						
Responds to name	Emotions readable	Interest in a variety of objects		Strings sounds together		
Follows point to object	Eye contact while playing	Uses objects appropriately^*∗*^		Variety of consonant sounds		
Requests object	Shared affect	Repeats simple activity over and over		Comprehension vocabulary		
Words for attention or help	Seeks attention			Block or ring stacking		
Points to objects	Gets you to laugh					
Waves to greet	Shows object for social attention					
	Initiating through handing object					
	Initiates joint attention					

*First-Year Inventory (FYI)*						
Looks when name is called		Upset when switching activities	Overly sensitive to touch	Babbles	Sleeping and waking patterns are regular	
Trouble hearing	Avoids looking at you	Enjoys staring at bright lights	Ignores loud or startling sounds	Uses pincer grip	Regular feeding patterns	
Looks at people when they talk	Looks at your face for comfort	Gets stuck on playing with a part of a toy	Spits out certain textures of foods	Eyes line up when looking at object		
Uses communicative gestures	Plays alone for an hour or more	Body gets stuck in positions or postures	Presses against things			
Responds to ‘‘Where's —?”	Looks up from play when shown new toy	Enjoys making objects spin over and over	Enjoys rubbing or scratching objects			
Uses finger to point at things	Easy to understand baby's expressions	Stares at fingers while wiggling them	Enjoys kicking feet over and over			
Plays or communicates less than in the past	Tries to get your attention to show things, or for interactive or physical games					
	Imitates mouth sounds, body movements, or movements, with objects					
	Tries to get attention by sound and gaze					
	Seems interested in other babies					

**Table:** (b) Level 2 ASD screening instruments

Social communication		Repetitive behaviors	Sensory	Developmental	Reactivity/temperament	Atypical information processing	Adaptive skills
Communication	Social						
*Autism Observation Scale for Infants (AOSI)*							
Eye contact		Atypical motor behavior	Atypical sensory behavior	Motor control	Behavioral reactivity	Visual tracking	
Orientation to name	Differential response to facial emotion	Insistence on specific objects/activities				Engagement of attention	
Social babbling	Anticipatory social response					Disengagement of attention	
Shared interest	Imitation					Transitions	
	Reciprocal social smile						
Coordination of eye gaze and action	Social interest and shared affect						

*Screening Test for Autism in Two-Year-Olds (STAT) (applied to children under 24 months)*							
Requests when presented with communication challenge (2 items)	Takes turns	(Lack of) functional object use^*∗*^		Functional object use^*∗*^			
Directs attention and interacts socially (4 items)	Imitates motor acts (4 items)						

*Parent Observation of Early Milestones Scale (POEMS)*							
Eye contact		Shifts (does not shift) attention from one event to another	Cuddling	Object permanence	Demands attention	Visual tracking (2 items)^*∗*^	Sleep (3 items)
Imitates sounds or words	Social smiling	Explores (does not explore) new toys and environments	Reaction to pain	Muscle tone	Stability of mood		Feeding (4 items)
Points to request	Laughs appropriately	Diverse toy play	Reaction to noise	Agility in movement	Comforted when hurt		
Points to share interests	Attachment to parents	Repetitive body movements	Fearful of objects that move or make noise	Attention span	Unpredictable reactions		
Coordinates gaze and point	Recognizes parents voice	Atypical use of words	Tolerance to transitions	Stacks blocks	Activity level		
Points in response to question	Emotions appropriate			Pretend play	Cries to express needs		
Follows adult point	Anticipation to being picked up			Communicates with words^*∗*^	Waiting tolerance		
Follows simple direction	Response to name			Visual tracking (2 items)^*∗*^			
Brings toys to request	Attention to faces						
Greets	Shifts attention from object to person						
Coordinates gestures and communication	Imitating						
Communicates with words^*∗*^	Social games						
	Interest in birthday present and cake						
	Interest in peers						
	Plays with peers						

*Autism Diagnostic Evaluation for Children (ADEC)*							
Responds to verbal command	Imitation	Functional play (lack of)	Responds to every day sounds	Demonstrates use of words			
Demonstrates use of words	Gaze switching from toy to person	Pretend play (lack of)					
Use of gestures (waves good-bye)	Eye contact						
Response to name	Reciprocity of smile						
	Following point						
	Anticipatory posture for being picked up						
	Nesting into caregiver						

^*∗*^Item placed in two categories.

**Table 7:** Methodological recommendations for future early autism screening studies.

Domain	Essential study features for interpretability: general for disability and autism screening	Additional criteria for interpretability for screening that targets children under 18 months	Recommended for consistency across studies
Sample/participants	Level 1: large population studies needed; track at least a subset of representative screen negatives to determine true and false negatives so that accurate Se and Sp can be calculated. Report demographic characteristics.		Report inclusion/exclusion criteria related to other risks or other disabilities; consider % of preterm/low birthweight; suggest to exclude significant sensory, motor, and known genetic diagnoses.
	Level 2: smaller (not population level) high-risk samples acceptable. Level 2: include samples matched on cognitive level or compare DD to ASD at outcome. Report demographic characteristics.	Level 2: include samples matched on cognitive level and/or compare DD to ASD on outcomes.	Report inclusion/exclusion criteria related to other risks or other disabilities; see above.

Screening instrument	Make administration of instrument as close as possible to how it would be used in a community setting.		

Reference standard	Evaluations based on DSM criteria; in-person BED preferable.		
	Evaluators must be blind to screening status of participants.		
	24-month-old diagnoses may be unstable in a minority of children; 36-month diagnosis is more reliable.	24-month-old diagnoses may be unstable in a minority of children; 36-month diagnosis is more reliable.	
	Include and define other disability outcomes.		Define other disability outcomes consistently; consider global delay, language delay, typical development, and other disabilities.

Timing and flow	Demonstrate a “clear path” between screening outcomes and scores and reference standard diagnosis.		
	Describe attrition.		
	No study to date has followed children up for diagnosis after the concurrent study age or at age of 3 years. It will be important for future studies to confirm longer-term diagnostic status.		

Evaluation and performance	Important to examine age groups separately; preferably in 6-month groupings up to 3 years of age.	Very important to examine young age groups separately, especially below 12 months versus 12–18 months versus 18–24 months.	Very important to examine young age groups separately, especially below 12 months versus 12–18 months versus 18–24 months.
	Very important to describe developmental level of children detected with screener.		Very important to describe developmental level of children detected with screener.
	Account for different levels of ASD severity.		
	Describe other disability outcomes for false positives.		
